# The landscape of emerging ceRNA crosstalks in colorectal cancer: Systems biological perspectives and translational applications

**DOI:** 10.1002/ctm2.153

**Published:** 2020-08-14

**Authors:** Xin Qi, Yuxin Lin, Jiajia Chen, Bairong Shen

**Affiliations:** ^1^ School of Chemistry, Biology and Material Engineering Suzhou University of Science and Technology Suzhou 215011 China; ^2^ Center for Systems Biology Soochow University Suzhou 215006 China; ^3^ Department of Urology The First Affiliated Hospital of Soochow University Suzhou 215006 China; ^4^ Institutes for Systems Genetics, West China Hospital Sichuan University Chengdu 610041 China

Dear Editor,

Colorectal cancer (CRC), a major gastrointestinal cancer with high prevalence, is seriously threatening the lives and health of millions of people around the world. Over the past decade, competing endogenous RNA (ceRNA) crosstalks that connect coding and noncoding RNAs (ncRNAs) through shared miRNAs have emerged as a novel layer of gene regulation. Here, we explore the activity of ceRNA crosstalk in oncogenic pathways regulating CRC cellular functions and highlight their promising potentials as biomarkers and therapeutic targets in CRC. Moreover, we summarize the current computational and experimental tools for ceRNA exploration and discuss the future perspectives to facilitate the translation of ceRNA research achievements into clinical applications.

Recent advances in high‐throughput sequencing technologies contribute to the extensive study of genomics and transcriptomics at unprecedented resolution, which unraveled that ncRNAs constitute the majority of human transcriptome.^1^ Especially, increasing works in the past decade have revolutionized our perception of ncRNAs from transcriptional "noise" to pivotal regulatory molecules that mediate a wide variety of cellular processes including transcriptional regulation, chromatin remodeling, and signal transduction.^2^ With innovative efforts to decipher how ncRNAs exert functions under physiological and pathological conditions, the ceRNA hypothesis has been proposed in 2011 by Salmena et al.^3^ It states that endogenous coding and noncoding transcripts sharing common miRNA response sequences (MREs) can indirectly regulate the expression of each other through competition for miRNA binding^3^ (Figure 1A). Under normal developmental and physiological conditions, ceRNAs are critical regulators of multiple processes, such as the differentiation of embryonic stem cells and skeletal muscle myogenesis. Currently, it has become increasingly clear that perturbation of ceRNA crosstalks profoundly contributes to CRC pathogenesis by mediating cell proliferation, migration, invasion, and apoptosis via diverse pivotal signaling pathways, including Wnt/β‐catenin, TGF‐β, PI3K/Akt/mTOR, EGFR1/RAF1/MAPK, and p53 signaling pathways (Figure 1B, Table S1).

**FIGURE 1:**
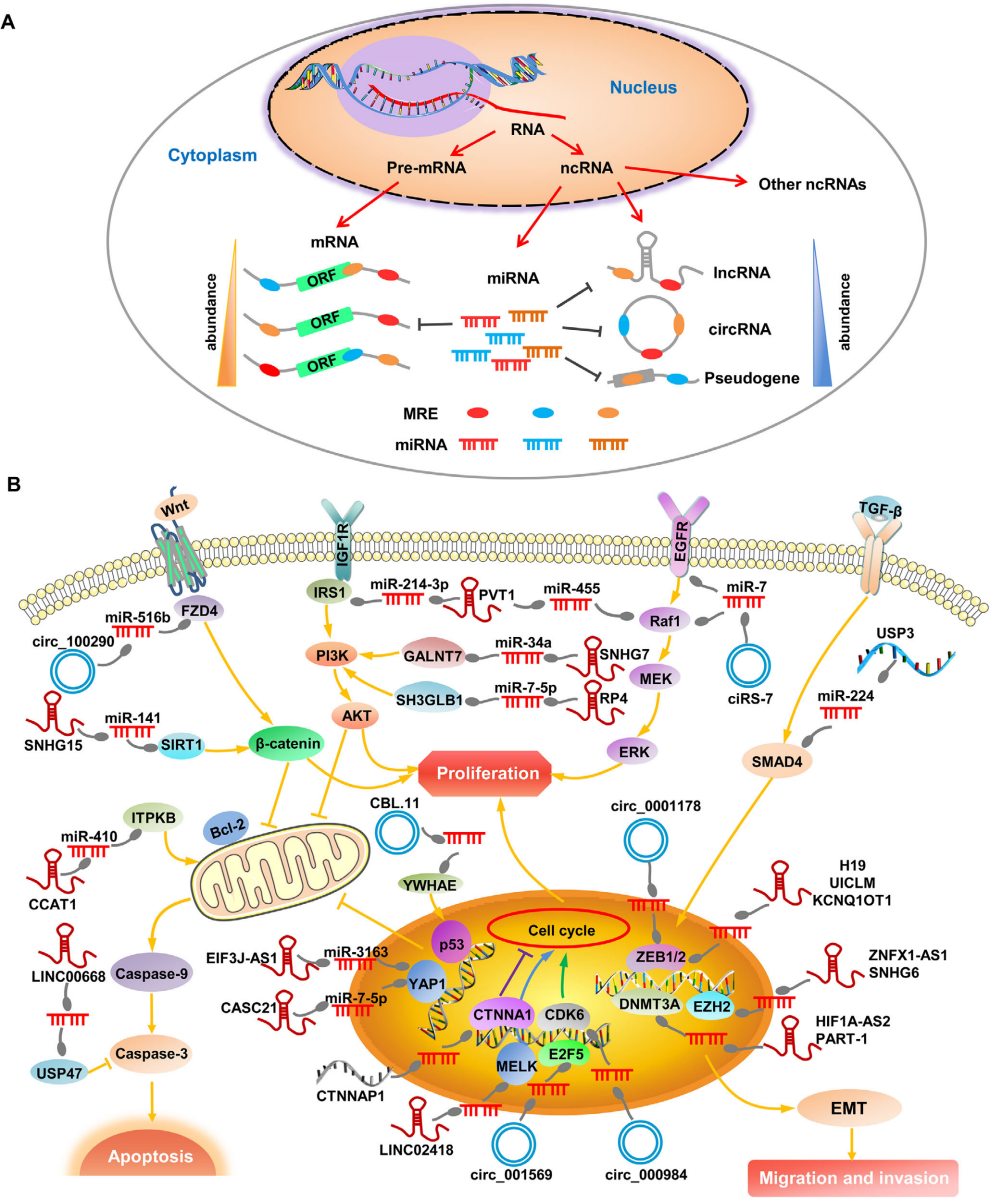
Schematic diagram of ceRNA mechanism and ceRNA crosstalks function in regulating CRC pathogenesis. A, According to the potential of encoding proteins, RNAs can be classified into mRNA and ncRNAs. Among them, ncRNAs contain multiple RNA types including miRNA, lncRNA, circRNA, pseudogene, and other ncRNAs. In particular, endogenous transcripts with shared MREs, such as lncRNA, circRNA, pseudogene, and mRNA, can reciprocally regulate each other's expression by competing for common miRNA binding via the ceRNA mechanism. B, ceRNA crosstalk has emerged as a key regulator of CRC cell proliferation, migration, invasion, and apoptosis by affecting a variety of signaling pathways including Wnt/β‐catenin, TGF‐β, PI3K/Akt/mTOR, EGFR1/RAF1/MAPK, and p53 signaling pathways.

As the research hotspots in ceRNA family, lncRNAs and circRNAs have increasingly emerged as biomarkers for CRC diagnosis, facilitating early detection, risk stratification of patients, and clinical decision making (Figure 2). Especially, given the important role of exosomes in intercellular communication, the detection of ceRNAs (e.g., LINC02418, circHIPK3, and UCA1) in serum exosomes opens up an innovative RNA‐based diagnostic strategy for liquid biopsy of tumors.^4^ Moreover, to improve clinical outcome through precise therapeutic targeting is one of the important goals of CRC management. Mounting ceRNA players (e.g., UICLM and circHIPK3) have emerged as promising prognostic biomarkers and molecular therapeutic targets for CRC patients due to their critical roles in tumorigenesis and/or tumor progression.^5^, ^6^ Furthermore, chemotherapeutic intervention after surgical resection is one of the most common treatment strategies for patients with advanced CRC. However, the acquired resistance to chemotherapeutic drugs including 5‐fluorouracil (5‐FU), oxaliplatin, and methotrexate is a major obstacle for the effective treatment of CRC. It has been demonstrated that lncRNA TUG1 mediates CRC resistance to methotrexate and 5‐FU via different ceRNA mechanisms^7^, ^8^ (Figure 2).

**FIGURE 2:**
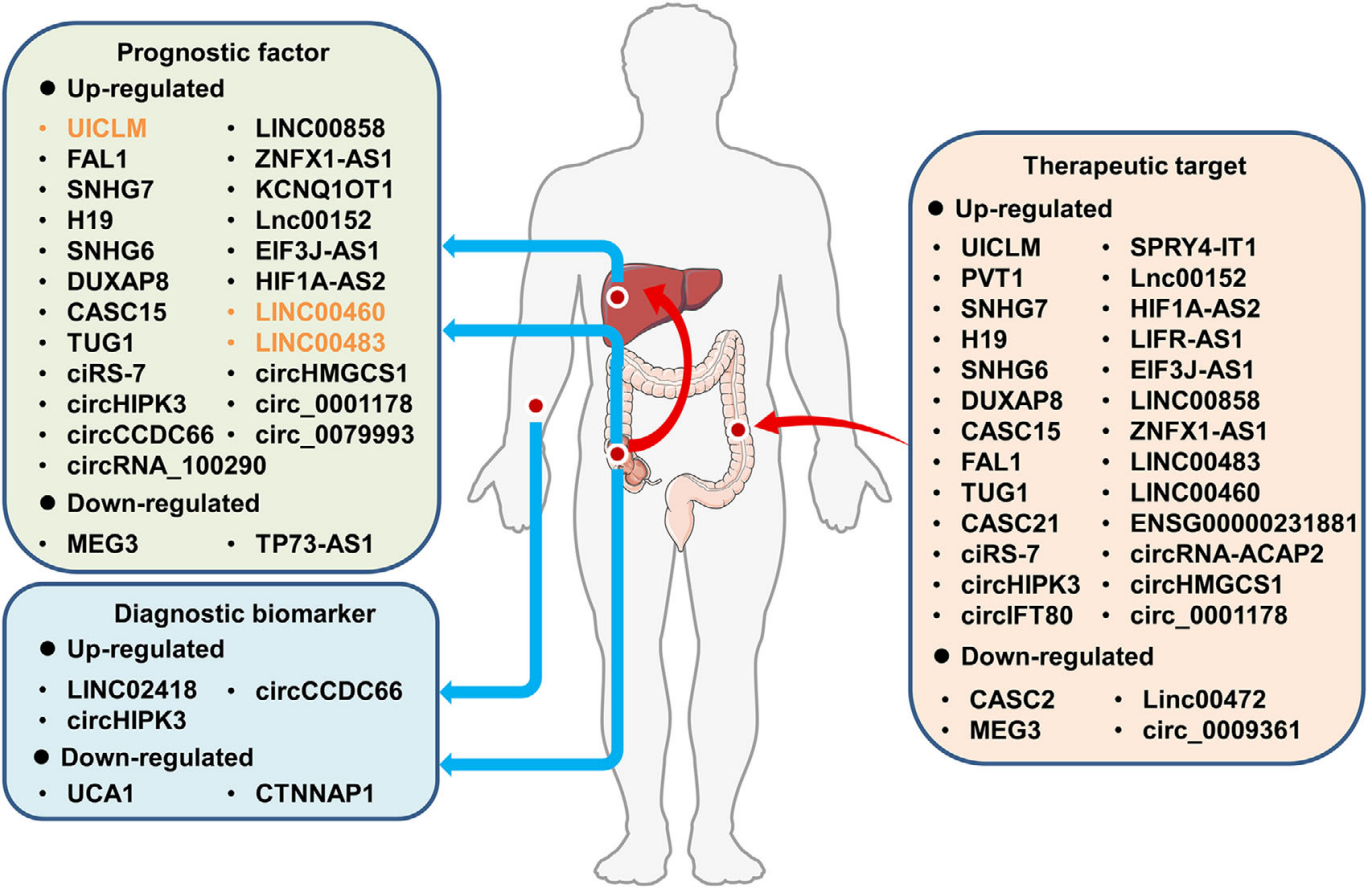
Translational implications of ceRNA molecules in CRC diagnosis, prognosis, and treatment. ceRNA molecules derived from serum exosomes (e.g., LINC02418, circHIPK3, and UCA1) or colorectal tissues (e.g., circCCDC66 and CTNNAP1) have been identified as promising diagnostic biomarkers for CRC patients. Multiple lncRNAs and circRNAs acting as ceRNAs have been shown to be closely related to tumor size, high‐grade tumors, metastasis, and survival time, exhibiting huge potential as prognostic biomarkers for CRC patients. For example, yellow‐labeled UICLM, LINC00460, and LINC00483 were reported to be closely related to liver metastasis of CRC. Moreover, a variety of ceRNA players have become attractive therapeutic targets for CRC patients due to their pivotal roles in CRC carcinogenesis and/or treatment outcomes.

Given the emerging pivotal roles of ceRNA crosstalks in cancer, substantial efforts have been made to develop computational and experimental approaches, contributing to the system‐level exploration of ceRNA interactions. Currently, approaches (e.g., prediction algorithms and high‐throughput RNA‐RNA interaction assays) used for identification of miRNA binding sites on target genes can be applied to discover ceRNA interplays. Notably, multiple ceRNA databases have been established to facilitate ceRNA identification. Based on the regulatory information, the present ceRNA relevant databases mainly contain two types: (1) binary regulatome providing miRNA‐target interactions; (2) triple regulatome containing ceRNA interactions (Table S2). Moreover, the direct binding between miRNA and ceRNA targets could be verified through in vitro (e.g., miRNA pull‐down assay and luciferase reporter assay) and in vivo experiments (e.g., RNA immunoprecipitation [RIP], gene overexpression, and knockdown).

Despite great progress has been achieved in decoding ceRNA landscapes involved in CRC carcinogenesis, the identity and function of most ceRNA players remain to be explored. Notably, besides the classical model of adenoma‐carcinoma sequence, the development process of CRC involves alternative pathways (e.g., de novo pathway, serrated pathway, and colitic cancer pathway), which can be defined on the basis of molecular features including DNA microsatellite instability, chromosomal instability, and CpG island methylator phenotype.^9^ However, the current research on ceRNA crosstalks do not involve specific subtypes of CRC, which have great impact on the clinical management of CRC prevention and treatment. Furthermore, it is critical to realize that various ceRNA crosstalks together constitute a complicated network, contributing to the fine regulation of CRC pathogenesis. Due to miRNA target multiplicity, a ceRNA molecule could participate in a variety of cancer‐related processes by sponging distinct miRNAs, while multiple ceRNAs may jointly control the dysregulation of a biological process through modulating the same target genes of different miRNAs. Therefore, the association of ceRNA with specific CRC subtypes and the sophisticated crosstalk between distinct ceRNAs should be considered when designing clinical strategies for CRC patients.

Furthermore, due to the multilevel complexity of ceRNA crosstalks, whether those endogenous RNAs act as *bona fide* ceRNAs under physiological conditions of the cell is a fundamental question to be progressively solved. Cancer is being regarded as a systems biology disease, which exhibits considerable complexity in regulatory circuits and molecules crosstalk rather than the dysregulation of individual gene or pathway. Therefore, the paradigm of “components→networks→interacting models→phenotype” is the future clue for system‐level ceRNA biomarker discovery in CRC. Compared with network biomarker, dynamic network biomarker has remarkable advantages in monitoring real‐time changed disease state and providing predictive predisease signals.^10^ Therefore, constructing dynamic and personalized ceRNA network and further mechanism and translation research are required to move the development of ceRNA‐based diagnostic, prognostic, and therapeutic approaches forward to benefit CRC patients.

In summary, ceRNA crosstalk is emerging as a novel layer of gene regulation that is tightly implicated in the hallmarks of CRC. In the present study, the decoding of pathological implications, translational potentials, and future directions of ceRNA crosstalks in CRC not only highlight new perspectives for CRC carcinogenesis but also expand our understanding of gene regulatory networks and illuminate new avenues to explore innovative strategies for CRC management.

## AUTHOR CONTRIBUTIONS

Xin Qi and Bairong Shen designed the manuscript. Xin Qi collected the related data and drafted the manuscript. Bairong Shen, Xin Qi, Yuxin Lin, and Jiajia Chen revised the manuscript. All authors read and approved the final manuscript.

## CONFLICT OF INTERESTS

The authors declare that there is no conflict of interest.

## Supporting information

Supporting Information
