# Supplementary material for: The landscape of emerging ceRNA crosstalks in colorectal cancer: Systems biological perspectives and translational applications
Source: Clin Transl Med. 2020 Aug 14;10(4):e153. doi: 10.1002/ctm2.153 (PMC7426901; doi:10.1002/ctm2.153)
Supplement: Supplementary file 1 — Supporting Information [file CTM2-10-e153-s001.docx]

**Table S1. Biological roles of literature-reported ceRNAs in CRC**

| **ceRNA** | **Shared miRNA** | **Competing Target** | **Biological function** | **Downstream pathway** | **PMID** |
| --- | --- | --- | --- | --- | --- |
| **lncRNA** |  |  |  |  |  |
| H19 | miR-138 and miR-200a | Vimentin, ZEB1, and ZEB2 | Promote EMT progression | EMT, TGF-β1 | 26068968 |
| H19 | miR-194–5p | SIRT1 | Mediate resistance to 5-fluorouracil | autophagy | 30451820 |
| SNHG3 | miR-182-5p | c-Myc | Promote proliferation |  | 28731158 |
| SNHG6 | miR-760 | FOXC1 | Promote proliferation, invasion and migration |  | 30254467 |
| SNHG6 | miR-26a-5p | ULK1 | Mediate resistance to 5-fluorouracil | autophagy | 31516391 |
| SNHG6 | miR-26a/b and miR-214 | EZH2 | Promote migration and invasion |  | 30626446 |
| SNHG7 | miR-34a | GALNT7 | Promote proliferation, migration and invasion and inhibit apoptosis | PI3K/Akt/mTOR | 29970122 |
| SNHG15 | miR-141 | SIRT1 | Promote proliferation, migration, invasion and inhibit apoptosis | Wnt/β-catenin | 31213086 |
| SNHG16 | NA | SCD | Promote migration and inhibit apoptosis | lipid metabolism | 27396952 |
| CASC2 | miR-18a | PIAS3 | Inhibit proliferation | STAT3 | 27198161 |
| UICLM | miR-215 | ZEB2 | Promote migration and invasion | EMT | 29187907 |
| PART-1 | miR-143 | DNMT3A | Promote proliferation and metastasis |  | 28619512 |
| SPRY4-IT1 | miR-101-3p | NA | Promote migration and invasion | EMT | 28720069 |
| CCAT1 | miR-410 | ITPKB | Inhibit proliferation and promote apoptosis |  | 29190961 |
| TUG1 | miR-186 | CPEB2 | Mediate methotrexate resistance |  | 28302487 |
| TUG1 | miR-197-3p | TYMS | Mediate resistance to 5-fluorouracil |  | 31528224 |
| TUSC7 | miR-211 | CDK6 | Inhibit proliferation |  | 28214867 |
| UCA1 | miR-135a, miR-143, miR-214, and miR-1271 | ANLN, BIRC5, IPO7, KIF2A, and KIF23 | Promote migration |  | 30195762 |
| PVT1 | miR-455 | RUNX2 and RAF1 | Promote proliferation, migration and invasion |  | 29637007 |
| PVT1 | miR-214-3p | IRS1 | Promote proliferation, invasion and inhibit apoptosis | PI3K/Akt | 31125260 |
| lnc00152 | miR-206 | NRP1 | Promote proliferation and invasion | EMT | 29956750 |
| HIF1A-AS2 | miR-129-5p | DNMT3A | Promote cell proliferation, invasion | EMT | 29278853 |
| LIFR-AS1 | miR-29a | TNFAIP3 | Mediate resistance to photodynamic therapy |  | 29807108 |
| RP4 | miR-7-5p | SH3GLB1 | Inhibit proliferation and promote apoptosis | autophagy and PI3K/Akt | 29531464 |
| MEG3 | miR-141 | PDCD4 | Mediate sensitivity to oxaliplatin |  | 30119236 |
| lincRNA-ROR | miR-145 | Oct4, Sox2, and Nanog | Promote proliferation and mediate sensitivity to chemotherapy |  | 29690669 |
| Linc00472 | miR-196a | PDCD4 | Inhibit proliferation and promote apoptosis |  | 29930217 |
| KCNQ1OT1 | miR-217 | ZEB1 | Promote migration | EMT | 30794031 |
| DUXAP8 | miR-577 | RAB14 | Promote migration and invasion |  | 31364111 |
| CACS15 | miR-145 | ABCC1 | Mediate resistance to oxaliplatin |  | 30639170 |
| EIF3J-AS1 | miR-3163 | YAP1 | Promote proliferation and inhibit apoptosis |  | 31709617 |
| GAS5 | miR-222-3p | PTEN | Inhibit migration and invasion and promote autophagy |  | 31400607 |
| DSCAM-AS1 | miR-144-5p | CDKL1 | Promote proliferation, migration and invasion |  | 31730864 |
| LINC00858 | miR-22-3p | YWHAZ | Promote proliferation, migration and invasion |  | 30931636 |
| ZNFX1-AS1 | miR-144 | EZH2 | Promote proliferation, migration and invasion |  | 30770796 |
| FAL1 | miR-637 | NUPR1 | Promote proliferation, migration and invasion |  | 30267804 |
| MALAT1 | miR-363-3p | EZH2 | Promote proliferation |  | 30972996 |
| LINC00668 | miR-188-5p | USP47 | Promote proliferation, migration and inhibit apoptosis |  | 31233752 |
| LINC00483 | miR-204-3p | FMNL2 | Promote proliferation and metastasis |  |  |
| XIST | miR-133a-3p | RhoA | Promote invasion | ROCK | 30678736 |
| LINC00460 | miR-939-3p | LIMK2 | Promote migration and invasion |  | 30863183 |
| LINC02418 | miR-1273g-3p | MELK | Promote proliferation and inhibit apoptosis | cell cycle | 31358735 |
| CASC21 | miR-7-5p | YAP1 | Promote proliferation, migration, invasion and inhibit apoptosis | EMT | 31731190 |
| ENSG00000231881 | miR-133b | VEGFC | Promote proliferation and metastasis |  | 30581003 |
| **circRNA** |  |  |  |  |  |
| ciRS-7 | miR-7 | EGFR and RAF1 | Promote proliferation, migration and invasion | MAPK | 28174233 |
| hsa_circ_000984 | miR-106b | CDK6 | Promote proliferation, migration and invasion |  | 29207676 |
| circRNA_100290 | miR-516b | FZD4 | Promote migration and invasion | Wnt/β-catenin | 30173892 |
| circRNA-ACAP2 | miR-21-5p | Tiam1 | Promote proliferation, migration and invasion |  | 30212824 |
| circHIPK3 | miR-7 | FAK, IGF1R, EGFR, YY1 | Promote proliferation, migration, invasion and inhibit apoptosis |  | 29549306 |
| circHMGCS1 | miR-503-5p | NA | Promote proliferation |  |  |
| circIFT80 | miR-1236-3p | HOXB7 | Promote invasion and migration | EMT | 31648103 |
| circ 0009361 | miR-582 | APC2 | Inhibit migration and invasion | EMT | 31109967 |
| CBL.11 | miR-6778-5p | YWHAE | Inhibit proliferation | p53 | 31438886 |
| circFMN2 | miR-1182 | hTERT | Promote proliferation and migration |  | 31738400 |
| circ_0079993 | miR-203a-3p.1 | CREB1 | Promote proliferation |  | 31515467 |
| circ_0001178 | miR-382, miR-587, miR-616 | ZEB1 | Promote migration and invasion | EMT | 31747371 |
| circRNA_001569 | miR-145 | E2F5, BAG4 and FMNL2 | Promote proliferation and invasion |  | 27058418 |
| circCCDC66 | miR-33b and miR-93 | MYC | Promote proliferation, migration and invasion |  | 28249903 |
| **pseudogene** |  |  |  |  |  |
| CTNNAP1 | miR-141 | CTNNA1 | Inhibit proliferation | G0/G1 cell cycle arrest | 27487124 |
| **mRNA** |  |  |  |  |  |
| USP3 | miR-224 | SMAD4 | Inhibit migration and invasion |  | 28655924 |
| ZNF148 | miR101, miR144, miR335, and miR365 | TOP2A | Inhibit proliferation |  | 28072746 |

**Table S2. Publicly available databases for ceRNA research**

| **Name** | **Description** | **URL** | **PMID** |
| --- | --- | --- | --- |
| **Binary regulatome** |  |  |  |
| starBase v2.0 | - A database providing the comprehensive miRNA-target interactions supported by CLIP-Seq | http://starbase.sysu.edu.cn/ | 24297251 |
| miRTarBase | - A curated database for experimentally supported miRNA-mRNA interactions | http://miRTarBase.mbc.nctu.edu.tw/ | 29126174 |
| DIANA-TarBase v8 | - A curated database providing a large number of experimentally verified miRNA-mRNA interactions | http://www.microrna.gr/tarbase/ | 29156006 |
| DIANA-LncBase v3 | - A manually curated database of experimentally supported miRNA-lncRNA interactions | www.microrna.gr/LncBase/ | 31732741 |
| miRSponge | - A manually curated database containing experimentally validated miRNA sponges and ceRNAs | http://www.bio-bigdata.net/miRSponge/ | 26424084 |
| CircInteractome | - A web tool for searching miRNA-binding sites on circRNAs | http://circinteractome.nia.nih.gov/ | 26669964 |
| CSCD | - A database for predicting mRNA response element sites on cancer-specific circRNAs | http://gb.whu.edu.cn/CSCD | 29036403 |
| SomamiR 2.0 | - A database of cancer somatic mutations affecting miRNA-mRNA, miRNA-circRNA and miRNA-lncRNA interactions | http://compbio.uthsc.edu/SomamiR/ | 26578591 |
| **Triple regulatome** |  |  |  |
| miRSponge | - A manually curated database containing experimentally validated miRNA sponges and ceRNAs | http://www.bio-bigdata.net/miRSponge/ | 26424084 |
| lnCeDB | - A database of potential lncRNAs acting as ceRNAs in human | http://gyanxet-beta.com/lncedb/ | 24926662 |
| LncACTdb 2.0 | - A manually curated database of lncRNA-associated ceRNAs supported by experiments | http://www.bio-bigdata.net/LncACTdb/ | 30476305 |
| CircNet | - A database providing tissue-specific circRNA expression pattern and circRNA-associated ceRNAs from RNA-Seq data | http://circnet.mbc.nctu.edu.tw/ | 26450965 |
| LnCeVar | - A curated database of genomic variations that disturb lncRNA-associated ceRNA interactions | http://www.bio-bigdata.net/LnCeVar/ | 31617563 |
